# Emotional Eating and Binge Eating Disorders and Night Eating Syndrome in Polycystic Ovary Syndrome—A Vicious Circle of Disease: A Systematic Review

**DOI:** 10.3390/nu15020295

**Published:** 2023-01-06

**Authors:** Ewelina Burnatowska, Agnieszka Wikarek, Paulina Oboza, Natalia Ogarek, Mateusz Glinianowicz, Piotr Kocelak, Magdalena Olszanecka-Glinianowicz

**Affiliations:** 1 Students’ Scientific Society at the Pathophysiology Unit, Department of Pathophysiology, Medical Faculty in Katowice, The Medical University of Silesia, 40-752 Katowice, Poland; 2 Pathophysiology Unit, Department of Pathophysiology, Medical Faculty in Katowice, The Medical University of Silesia, 40-752 Katowice, Poland; 3 Department of Psychology, Social Sciences, and Humanities, School of Health Sciences in Katowice, the Medical University of Silesia, 40-752 Katowice, Poland; 4 Health Promotion and Obesity Management Unit, Department of Pathophysiology, Medical Faculty in Katowice, The Medical University of Silesia, 40-752 Katowice, Poland

**Keywords:** emotional eating, binge eating disorder, obesity, depression, anxiety, polycystic ovary syndrome

## Abstract

Obesity is an established risk factor for the development of polycystic ovary syndrome (PCOS), especially phenotype A. PCOS is an important cause of fertility disorders in a large group of women of reproductive age. For many years, effective methods of treating hormonal disorders associated with PCOS have been sought in order to restore ovulation with regular menstrual cycles. Numerous studies support obesity treatment as an effective therapeutic method for many women. A seemingly simple method of treatment may prove to be particularly difficult in this group of women. The reason for this may be the lack of recognition the primary cause of obesity development or the occurrence of a vicious circle of disease. Primary causes of developing obesity may be emotional eating (EE) and eating disorders (EDs), such as binge eating disorder (BED) and its extreme form, addictive eating, as well as night eating syndrome (NES). All of these are caused by impaired function of the reward system. Consequently, these disorders can develop or be exacerbated in women with obesity and PCOS as a result of depression and anxiety related to hirsutism and fertility disturbances. Therefore, for the effective treatment of obesity, it is very important to recognize and treat EE, BED, and NES, including the appropriate selection of pharmacotherapy and psychotherapy. Therefore, the aim of our manuscript is to analyze the available data on the relationships between EE, BED, NES, obesity, and PCOS and their impact on the treatment of obesity in women with PCOS.

## 1. Introduction

**Figure 1:**
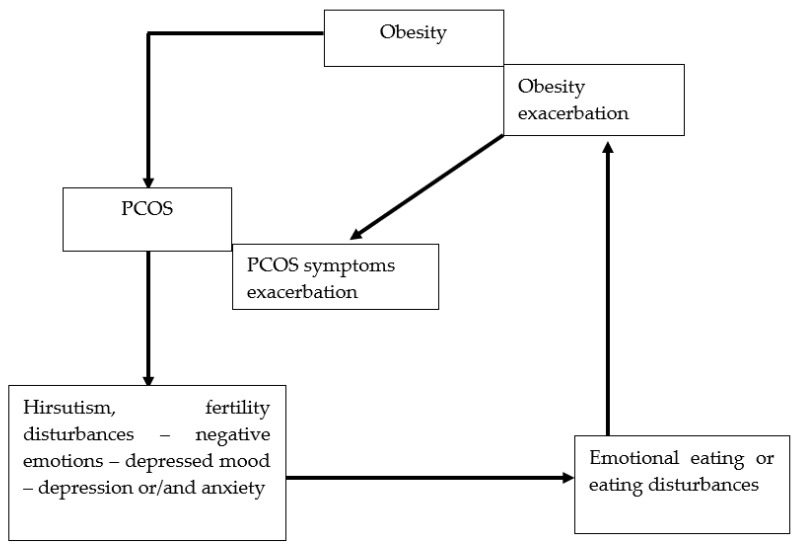
Vicious circle between EE or EDs and obesity and PCOS. EE: emotional eating; EDs: eating disorders; PCOS: polycystic ovary syndrome.

In 1935, Stein and Leventhal described a syndrome that included obesity, menstrual disturbances, enlargement of the ovaries, hirsutism, and sterility [1]. Today this syndrome is called polycystic ovary syndrome (PCOS), and it is known that obesity is the cause, not a symptom [2]. Treatment of obesity is a recognized method of PCOS therapy [3]. However, effective treatment of obesity requires proper diagnosis of the cause, including EE and EDs, such as BED, and its extreme form, addictive eating, as well as NES. [4]. All of these are caused by impaired function of the reward system. [5]. It should be noted that obesity is the cause of PCOS, but PCOS symptoms such as hirsutism and fertility disturbances may be a cause of subsequent depression and anxiety, which impair the function of the reward system and cause or exacerbate EE, BED, and NES, which in turn is the cause of obesity and exacerbation of hormonal disturbances. This creates a vicious circle of disease (Figure 1). Recognizing and treating EE, BED, and NES is very important to break this cycle [6].

Obesity induces the development of PCOS in genetically predisposed women [2]. The newest hypothesis suggests that maternal obesity and stress are predisposing factors in-utero to the development of EDs and PCOS during adolescence. Furthermore, chronic psychological stress related to low self-esteem, problems at school or/and home, and discrimination during puberty or adolescence may also be the factors that induce EE and, subsequently, BED. Moreover, chronic stress related to increased activity of the hypothalamic–pituitary–adrenal axis stimulates adrenal androgen synthesis [7]. Depression and anxiety may be linked to EE and BED as well as subsequent obesity, leading to the complication of PCOS. Numerous studies have shown an increased prevalence of depression, anxiety, and post-traumatic stress among children and adolescents during the COVID-19 pandemic. Therefore, an increase in the incidence of EE and BED can reasonably be expected [8]. This, in turn, may be the cause of the increase in the incidence of obesity in adolescents and the concurrent increase in the frequency of fertility disturbances in women of reproductive age associated with PCOS.

Thus, the purpose of this review was to summarize available data describing the relationships between EE, BED, NES, obesity, and PCOS. Moreover, we analyzed potential vicious cycle mechanisms between EE, BED, NES, obesity, and PCOS and the need to change the approach to the diagnosis and treatment of obesity in women with PCOS.

## 2. Methods

### 2.1. Search Strategy

**Figure 2:**
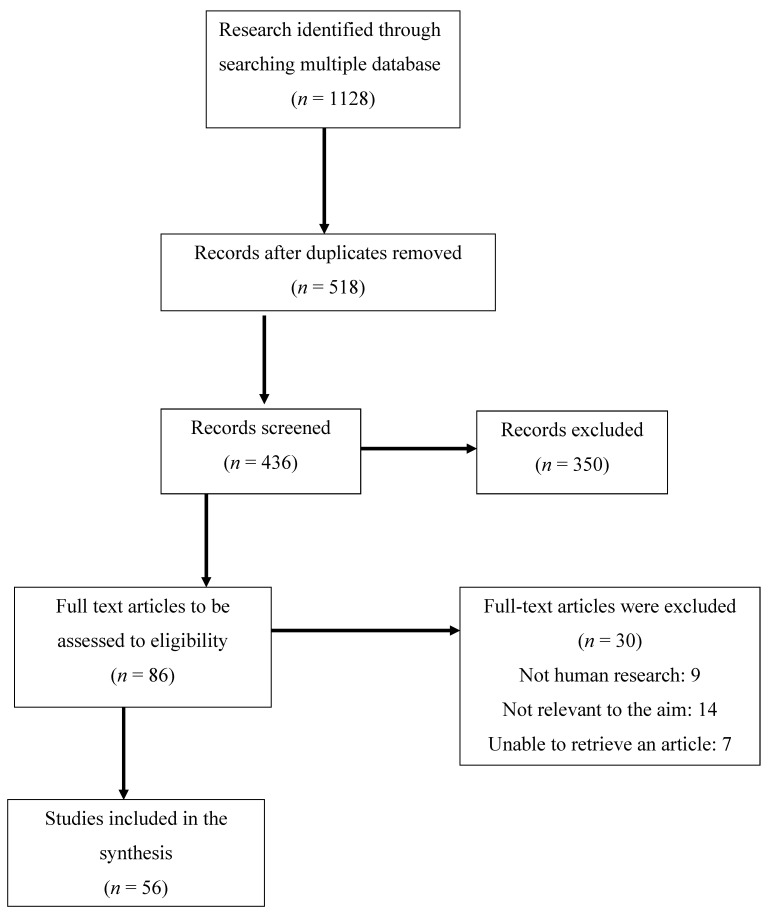
Flow chart of the proceedings in the selection of sources.

PubMed, Embase, Cochrane, and Web of Science databases were searched for related studies until 15 November 2022. A text search with the following keywords singly or in combination was conducted: ‘Emotional eating’, ‘Binge eating disorder’, ‘Night eating syndrome’, ‘Depression’, ‘Anxiety’, ‘Mental health’, ‘Obesity’, ‘PCOS’. The final search results were exported into EndNote, and duplicates were removed. The detailed search strategy is shown in Figure 2.

### 2.2. Inclusion and Exclusion Criteria 

Accepted studies met the following criteria: (1) analysis of mental health and/or emotional eating and eating disorders in PCOS women, (2) articles published in English, (3) studies involving human participants, and (4) studies including single measurements, longitudinal studies, and meta-analysis. Papers were excluded if they did not fit into the conceptual framework of the study. Studies including patients with mental illnesses other than depression and anxiety were also excluded.

### 2.3. Data Extraction

Data extraction was conducted with the following information: (1) name of the first author, (2) publication year, (3) country, (4) study design, (5) sample size, (6) PCOS diagnosis, (7) mental health assessment, (8) EE assessment, (9) BED and/or NES assessment, and (10) comparison with a control group. Due to the fact such a small number of studies were performed among PCOS women and our manuscript is a narrative review, the quality of the research was not assessed as a meta-analysis.

## 3. The Links between Brain Pathways of Food Intake Regulation and Endocrine Disturbances in PCOS

**Figure 3:**
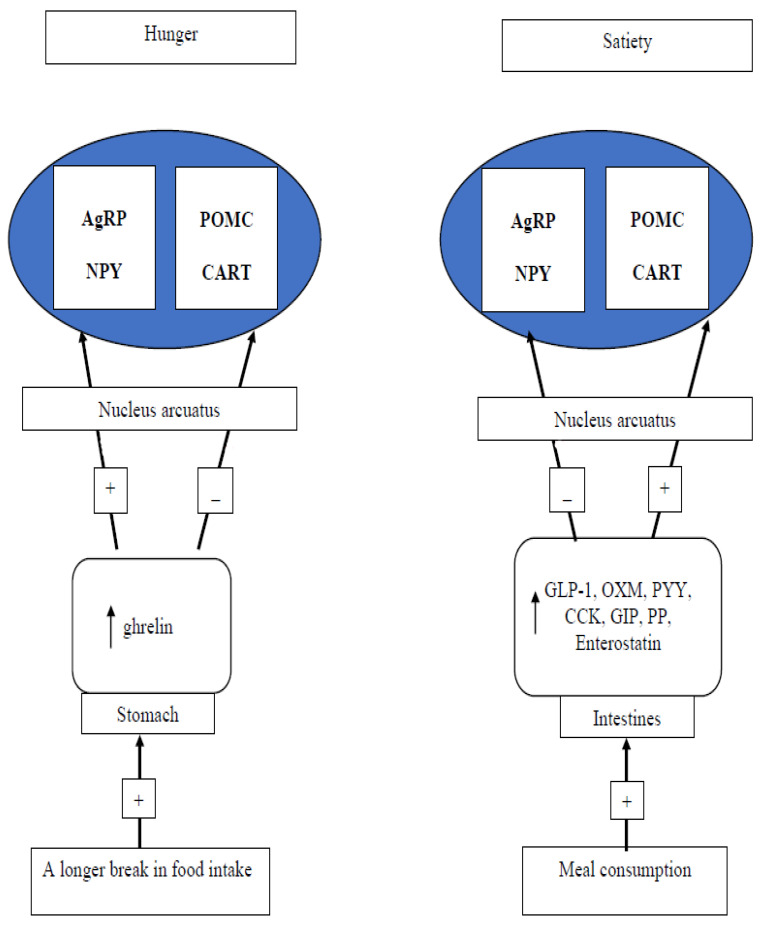
The brain–gut axis in the regulation of feeling hunger and satiety. AgRP: agouti-related peptide; NPY: neuropeptide Y; POMC: pro-opiomelanocortin; CART: cocaine- and amphetamine-regulated transcript; GLP-1: glucagon-like peptide 1; OXM: oxyntomodulin; PYY: peptide tyrosine tyrosine; CCK: cholecystokinin; GIP: glucose-dependent insulinotropic polypeptide; PP: pancreatic polypeptide.

Regulation of food intake and eating behavior is complex. Hunger and satiety regulation includes central centers of satiety and hunger feelings in the hypothalamus that receive hormonal signals from the digestive tract and adipose tissue (Figure 3). Moreover, the reward system (the amygdala/hippocampus, insula, orbitofrontal cortex (OFC), and striatum) is responsible for the regulation of food intake related to the feeling of pleasure (reward) and improving mood, called appetite or food craving. The main factor regulating this aspect of food intake is experienced emotions [9,10].

The key structures involved in emotion and reward value are the amygdala and OFC [11]. In turn, the hippocampus is a key structure responsible for episodic memory [12]. The function emotional and memory domains are integrated by the cingulate cortex. The cingulate cortex seems to be responsible for action–outcome learning, i.e., learning to obtain goals based on the outcomes, rewards, and punishments received for different actions [13]. The information related to a reward from the medial OCF and related to punishment and not receiving a reward from the lateral OCF is transferred to the pregenual anterior cingulate cortex. This information is used by the cingulate cortex for learning the action to perform to obtain a reward or avoid punishment (action–outcome learning). Thus, the anterior cingulate cortex linking reward and punishment information is related to emotion. Meanwhile, the posterior cingulate cortex transmits visuospatial information to the hippocampus where they are connected with the object and reward-related information to form episodic memory [14,15,16,17].

The main neurotransmitter associated with the action of reward is dopamine. The ventral tegmental area (dopaminergic nuclei of the tegmental area and substantia nigra, which contain the cell bodies of dopaminergic neurons) and the nucleus accumbens are where dopaminergic neurons send their axons (afferent mesolimbic pathway). The dopaminergic nuclei of the tegmental area also send axons to the amygdala (pleasure inhibition) and the lateral hypothalamus (pleasure stimulation). In turn, the substantia nigra sends signals to the striatum and nuclei of the forebrain. The dopaminergic nuclei of the tegmental area and the nucleus accumbens also send signals to the prefrontal cortex. Different parts of the prefrontal cortex activate or inhibit other structures of the brain depending on whether or not to perform a particular behavior. In this way, cognitive processes can control emotions and impulsive reactions [18].

The reward system is associated with the hypothalamus, and the integration of energy homeostasis and reward information occurs in the lateral hypothalamic nucleus, which modulates the functions of the ventral tegmental area and brainstem nucleus as well as the solitary tract nucleus responsible for modulation of intestinal signals and the feeling of satiety. Moreover, through CB1 receptors in the hypothalamus, the endocannabinoid system affects the functions of the arcuate nucleus and lateral hypothalamus, which communicate with the nucleus accumbens [19].

Three components of reward are described as liking, wanting, and learning, which are related to each other. Liking and wanting are responsible for the hedonic influence and reward motivation. In contrast, learning involves association and reward anticipation. Experimental studies conducted on animals have shown that endogenous opioids and the endocannabinoid system are responsible for the (hedonistic) aspect of liking, and dopamine is responsible for the willingness and learning aspect [20].

The function of the reward system is regulated by cortisol which inhibits dopamine release and, in vulnerable individuals, promotes EE [21]. The upregulation of the hypothalamic–pituitary–adrenal (HPA) action related to acute and chronic stress inhibits dopamine release in the reward system. This was confirmed in imaging studies with the use of functional magnetic resonance imaging (fMRI) [22,23]. In healthy women, estradiol and progesterone play an important role in the regulation of reward system function [24]. In the periovulatory period of the menstrual cycle, higher estradiol levels increase dopamine signaling in the reward system while increased progesterone secretion during the luteal phase decreases dopamine receptor sensitivity [25].

In PCOS women, excessive androgen levels may be a factor in increasing depressed mood and anxiety as well as appetite due to negative affective states and activation of HPA [26,27,28,29]. The results of some studies indicate the role of insulin resistance in the regulation of the reward system. 

One of them shows greater limbic activation in fMRI during an emotion task in insulin-resistant PCOS women compared to controls associated with mu-opioid binding potential. In addition, a four-month metformin treatment decreased the availability of these receptors [30]. In another study, the association between corticolimbic responses to pictures of food and insulin sensitivity was reported [31]. However, it should be noted that both these studies were performed in small groups and without adjustment for anthropometric parameters [30,31]. It is well-known that insulin resistance is the effect of visceral obesity, and obesity may be caused by EE and BED related to changes in neurotransmission in the reward system [32,33].

## 4. Emotional Eating—Definition, Mechanism, and Risk Factors in PCOS

EE, formerly called stress eating, is the propensity to eat in response to positive and negative emotions and not feeling hungry [34]. EE is a risk factor for the development of BED and is an extremely severe form of addictive eating. EE is a way to deal with emotions, but this strategy is not effective. Therefore, over time, it can develop into BED [35].

Numerous studies that assessed various aspects between personality traits and obesity have shown that impulsivity (tendency to act rapidly without consideration of consequences, including various psychological components such as disinhibition, neuroticism, extraversion, sensation seeking, inattention, impulsive decision making, insufficient inhibitory control, and lack of cognitive flexibility) is associated with high arousal responses to potential rewards and low level of self-control. All these factors in combination with the exhibition of an ‘obesogenic’ environment are common to the development of EE and BED and, consequently, obesity [36]. However, it should be noted that the main cause of EE seems to be the dysfunction of mesolimbic dopamine neurons in the reward system [5]. The changes in the function of the reward system in people with obesity were confirmed by fMRI [37,38,39]. Activation of the HPA axis by stress inhibits dopamine release in the reward/motivation system and slows down the inhibitory-control pathways [23].

An online survey including 455 women with PCOS (340 with obesity, 70 overweight, and 45 normal weight) showed that women with obesity and PCOS were characterized by high mean food cravings-trait scores compared to overweight and normal-weight women. In addition, multiple regression analysis found that craving-eating scores and EE scores were significant predictors of obesity [40]. Still, other data have shown that 30–50% of women with PCOS presented with EE [41]. There is a lack of other studies assessing the occurrence of EE in women with PCOS.

## 5. Eating Disorders (EDs)—Definition, the Type Associated with the Development of Obesity, Occurrence in Women with PCOS

EDs are a broad spectrum of mental disorders that considerably impair physical health and disrupt psychosocial functioning and include anorexia nervosa, bulimia nervosa, BED, avoidant–restrictive food intake disorder, pica, and rumination disorder [42]. NES is also included as an eating disorder.

BED, according to the fifth edition of the *Diagnostic and Statistical Manual of Mental Disorders* (DSM), is diagnosed if at least once per week for 3 months or more occurs the consuming of unusually large amounts of food in a short time with a loss of control. In addition, at least three of the following must be present: consuming food more rapidly than normal, eating until uncomfortably full, consuming large amounts of food without the feeling of hunger, eating alone to avoid shame or feeling disgusted with oneself, depression, or guilt after gluttony without any regular compensatory behavior [43].

**Figure 4:**
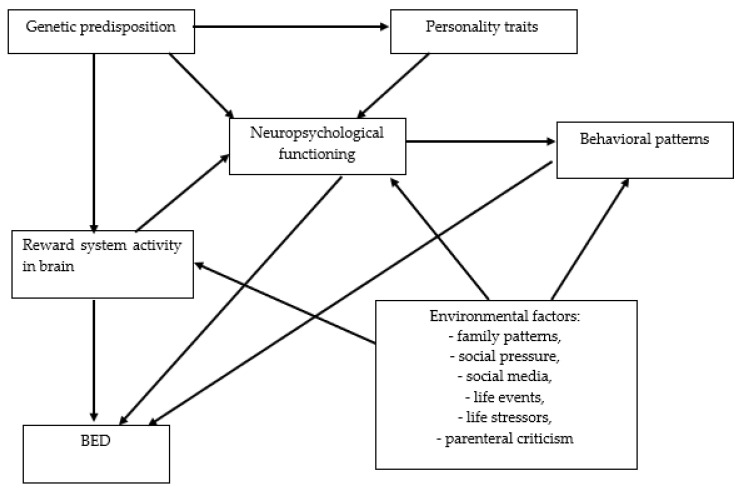
The network of relationships between risk factors for the development of BED. BED: binge eating disorder.

Two main subtypes of BED are distinguished: binge-first and diet-first [44]. The risk factors of the development of BED are sometimes divided into individual and environmental. The individual risk factors of BED development include genetic predisposition, personality traits, neuropsychological functioning, reward system activity in the brain, and behavioral patterns whereas, environmental risk factors include family patterns, social pressure, social media, life events, life stressors, parenteral criticism, and pregnancy stressors (Figure 4) [45].

The proposed diagnostic criteria for NES include recurrent episodes of excessive food consumption after dinner or eating after awakening from sleep and at the least three of the following: morning anorexia, a strong urge to eat between dinner and sleep and/or during the night, sleep onset and/or maintenance insomnia, frequently depressed mood or mood worsening in the evening, and a belief that one cannot get back to sleep without eating [46].

**Figure 5:**
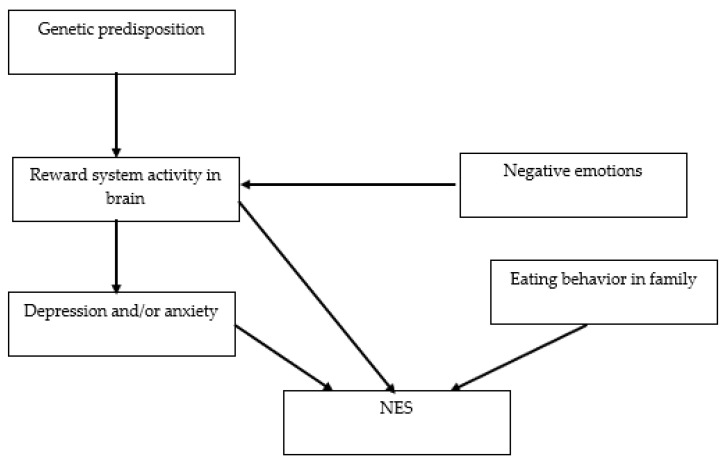
The network of relationships between risk factors for the development of NES. NES: night eating syndrome.

The risk factors for the development of NES include genetic predisposition, eating behavior in the family, negative emotions, depression, and anxiety (Figure 5) [47].

It is estimated that BED occurs in between 1–3% of the general population [48] and NES in 1.5% [49]. It should be noted that the association between BED and NES and the development of obesity was described many times [50]. The risk of the development of obesity among persons with BED is 3–6 times higher than in persons without BED [51]. In many cases, BED is the cause of the development of childhood obesity [52]. NES was diagnosed in 55% of persons seeking bariatric surgery [53]. It should be noted that in 15–20% of patients with NES, BED also occurs [54].

The association between BED or the history of BED and the occurrence of PCOS was described [55]. It was estimated there is a fourfold higher risk of development of BED in women with PCOS than in those without PCOS [56]. However, the survey performed during routine visits among 148 women with PCOS and 106 without showed the occurrence of BED in 17.6% and NES in 12.9% of women with PCOS without differences in frequency in the control group [57]. In turn, in another study, binge eating (BE) behavior occurred frequently in women with obesity compared to overweight and normal-weight women. Moreover, higher BE symptom scores were observed in the PCOS group than in normal-weight women without PCOS [40].

It should be noted that analysis of data describing EDs in PCOS women towards BED and NES is very difficult as a majority of studies in this group assessed EDs but did not provide these diagnoses and did not distinguish between BED and bulimia nervosa in compulsive behavior. Additional large studies are needed to determine the role of BED and NES in the development of PCOS. It would be particularly important to conduct observational studies over many years, including girls around puberty.

## 6. The Link between Depression and Anxiety and EE, BED, and NES in PCOS Women

A meta-analysis of 24 cross-sectional studies including 2316 women with PCOS showed an increased pool prevalence of depression (42%, 95% CI: 33–52%) and 16 cross-sectional studies including 1,698 women with PCOS showed an increased pool prevalence of anxiety (37%, 95% CI: 14–60%) [58]. A systemic review and meta-analysis studies including 30 cross-sectional studies involving 3050 women with PCOS and 3858 women as a control showed increased odds of symptoms of depression (OR: 3.78; 95% CI: 3.03–4.72; 18 studies), moderate/severe symptoms of depression (OR: 4.18; 95% CI: 2.68–6.52; 11 studies), any symptoms of anxiety (OR: 5.62; 95% CI: 3.22–9.80, 9 studies), and moderate/severe symptoms of anxiety (OR: 6.55; 95% CI: 2.87, 14.93; 5 studies) in women with PCOS. The factors associated with the increased prevalence of depression and anxiety were advanced age, higher BMI value, and hirsutism score [59]. In another analysis of six studies including 661 women (343 with PCOS and 318 without), the rate of subjects with symptoms of anxiety and depression was significantly higher in the PCOS group (OR = 2.76; 95% CI 1.26–6.02 and OR = 3.51; 95% CI 1.97–6.24) [60].

Mood disturbances in women with PCOS are the effect of negative emotions related to hyperandrogenism, infertility, and obesity. Chronic stress caused by negative emotions activated the HPA axis and increased cortisol secretion. Increased cortisol levels suppressed both dopamine and serotonin release [7,61]. As was mentioned above, the disturbances of neurotransmitters are compensated by food intake to regulate emotions such as in EE, BED, or NES [62]. It has been shown that 32% of subjects with BED had an episode of major depression and 12% of a general anxiety disorder [63]. The frequent occurrence of both depression and BED among Saudi Arabian women with (*n* = 116) compared to without (*n* = 378) PCOS was found [64].

In a population-based study including 10,000 participants aged 18–80 years, the results of multivariate regression analyses showed significant positive associations between anxiety and disinhibition as well as hunger [65]. In another study, the occurrence among subjects with BED of depression symptoms was 54.2%, anxiety 37.1%, and substance use 24.8% [66] while the results obtained in a population of 11,588 Swedish adults presenting to an Eating Disorder Treatment Clinic showed a reverse rate of anxiety and depression among women with BED (55% and 45%, respectively) and were more frequent than among women with anorexia nervosa and bulimia nervosa [67]. Only one study assessed the association between BED and depression and/or anxiety in PCOS women. This study showed that women with PCOS and concurrent anxiety symptoms have an increased risk of BE independent of obesity [57]. 

The Korea Community Health Survey which included 34,358 individuals aged 19 years and over revealed a higher depression score in subjects with episodes of NES and an association between levels of depression and NES [68]. Episodes of NES have been observed in 7.8% of patients with generalized anxiety disorder [69]. There is a lack of studies assessing any association between anxiety or depression and NES in PCOS. Further, studies are necessary to analyze possible associations.

## 7. It Is Possible to Effectively Treat Obesity in PCOS?

There are many myths surrounding the problems associated with the difficulties in treating obesity in women with PCOS. The main one is that PCOS is the cause of obesity, so patients see no point in treating obesity alone because of the perception that PCOS must be treated first. The second myth is that you need to treat insulin resistance, not obesity. Insulin resistance is the result of obesity, and the most effective way to improve insulin sensitivity is weight loss. It is also often wrongly believed that the inclusion of metformin in the therapy will result in weight reduction. A systemic review and meta-analysis of randomized trials showed a lack of efficacy of metformin in obesity treatment [70].

Low-efficiency obesity treatment related to feeling frustration and appetite has been shown [30]. As shown above, the main reason for the lack of effectiveness of obesity treatment in women with PCOS may be the lack of recognition of EE, BED, and NES [71,72]. Disturbances in the reward system prevent the long-term implementation of recommendations for lifestyle changes. Thus, screening for EE, BED, and NES should be performed during routine healthcare visits in women with PCOS [41,73]. It should be noted that this procedure should not only be limited to women diagnosed with obesity based on BMI but should be applied to all women with PCOS, which will help to prevent the development of obesity. Moreover, obesity should not only be diagnosed based on BMI but also based on waist circumference and body fat percentage [3]. One of the tools that can be used in the screening of ED diagnosis is the five-question SCOFF questionnaire [74] and in EE, the seven-question questionnaire developed by The Polish Association for the Study on Obesity [4]. These simple tools can be used in the office of every endocrinologist and gynecologist.

The first-line obesity treatment recommended for PCOS is lifestyle changes, including diet, exercise, and behavioral therapy [62]. However, it has been shown that the presence of BED increases the dropout rate in weight management programs [75]. It should be noted that recommendations for diet restriction and behavioral education may cause BED development in women with EE or BED exacerbation [76]. Therefore, in accordance with Polish [4] and Canadian [77] recommendations, in women with PCOS and EE, BED, or NES, if there are no contraindications, pharmacological treatment of obesity should be used, and the medications recommended in these patients is a combination of bupropion and naltrexone. In addition, psychotherapy, especially cognitive behavioral therapy (CBT), should be recommended [78].

## 8. The Limitation of the Review

The main limitation of the review is the lack of longitudinal and follow-up studies. The second limitation is that most of the studies were performed in small groups. Third, limited studies assessed the occurrence of BED and NES in PCOS, only a single study among binge eating behavior distinguished BED. Fourth, the association between the occurrence of depression or anxiety and EE, BED, or NES in women with PCOS has almost not been analyzed. Fifth, this review did not include publications in languages other than English.

## 9. Conclusions

Similar to bidirectional links between obesity and depression, the relation between EE, BED, and NES may also be bidirectional. Thus, EE, BED, and NES may be the primary cause of obesity development and its hormonal complication such as PCOS, but also EE, BED, and NES may develop in women with PCOS and cause or exacerbate the development of obesity and in consequence hormonal disturbances. The occurrence of EE, BED, and NES may be the cause of obesity treatment failure in women with PCOS. In turn, ineffective treatment of obesity may lead to growing frustration due to failure of attempts to become pregnant and the development or exacerbation of depression and/or anxiety and dealing with negative emotions with food. Thus, women with PCOS should be screened for EE, BED, and NES. In the treatment of obesity (not only increased BMI but also waist circumference and elevated body fat percentage) in women with PCOS and EE, BED, or NES, it is necessary to implement appropriately selected pharmacotherapy and psychotherapy. Changing the approach to the treatment of obesity in women with PCOS is especially important because the COVID-19 pandemic resulted in the deterioration of mental health in children and adolescents. This may result in an increased prevalence of EE, BED, NES, obesity, and fertility disturbances related to PCOS in the future.

It is necessary to carry out long-term observational studies assessing the impact of EE, BED, and NES on the development of obesity in adolescents and young women as well as their impact on fertility in the reproductive-age population. In addition, an important direction of research would be to observe how the proposed changes in the approach to diagnosing and treating obesity in women with PCOS affect the long-term improvement of their hormonal profile and the restoration of fertility.

## Data Availability

Not applicable.
